# Impaired psychological well-being of healthcare workers in a German department of anesthesiology is independent of immediate SARS-CoV-2 exposure – a longitudinal observational study

**DOI:** 10.3205/000298

**Published:** 2021-09-01

**Authors:** Benedikt Schmid, Stefan M. Schulz, Michael Schuler, Dennis Göpfert, Grit Hein, Peter Heuschmann, Thomas Wurmb, Paul Pauli, Patrick Meybohm, Heike L. Rittner

**Affiliations:** 1 Department of Anesthesiology, Würzburg University Hospital, Würzburg, Germany; 2 Department of Psychology I – Biological Psychology, Clinical Psychology and Psychotherapy, Julius Maximilians University, Würzburg, Germany; 3 Institute for Clinical Epidemiology and Biometry, Julius Maximilians University, Würzburg, Germany; 4 Department of Psychiatry, Würzburg University Hospital, Würzburg, Germany; 5 Clinical Trial Centre, Würzburg University Hospital, Würzburg, Germany

**Keywords:** anesthesia, critical care, COVID-19, healthcare workers, mental health

## Abstract

**Background:** The study aimed to assess the mental well-being of healthcare professionals at a German department of anesthesiology and critical care with a specialized ICU for treatment of COVID-19 patients during the first two peaks of the 2020 pandemic, and identifying risk and protective factors.

**Methods:** A single-center longitudinal, online-based survey was conducted in healthcare workers from a department of anesthesiology and critical care in Bavaria, the most affected federal state in Germany at the time of assessment. Validated scores for depression, anxiety, somatic disorders, burnout, resilience, and self-management were used and complemented by questions about perceived COVID-19-related stressors. In parallel, patient characteristics in the ICU were collected.

**Results:** 24 and 23 critically ill COVID-19 patients were treated during both observation periods in April/May and November/December 2020, respectively. 87.5% and 78.2% of patients had moderate to severe acute respiratory distress syndrome. From March 6, 2020 onwards, the hospital had switched to a command and control-based hospital incident command system (HICS) and increased work forces. Point prevalence of depression-like symptoms (13.6% and 12.8%) and burnout (21.6% and 17.4%) in the department’s healthcare professionals was high. Exposure to SARS-CoV-2 did not increase psychological burden. Consequences of the lockdown were rated as highly distressing by a majority of all ICU personnel. High self-reported trait resilience was protective against signs of depression, generalized anxiety, and burnout.

**Conclusions:** During the pandemic, healthcare professionals have been suffering from increased psychological distress compared to reference data for both the general population and ICU personnel. General effects of the lockdown appear more relevant than actual COVID-19 patient contact. High trait resilience has a protective effect, yet vulnerable individuals may require specific support. Prevention against potential after effects of the lockdown, and in particular measures allowing to avoid another lockdown, appear warranted.

## Zusammenfassung

**Hintergrund:** Die Studie hatte zum Ziel, die psychische Gesundheit der anästhesiologischen Mitarbeiterinnen und Mitarbeiter einer Universitätsklinik mit einer auf COVID-19 spezialisierten Intensivstation während der ersten beiden Wellen der Pandemie im Frühjahr und Herbst 2020 zu untersuchen und sowohl Risikofaktoren als auch protektive Faktoren zu identifizieren.

**Methoden:** Es wurde eine monozentrische, Web-basierte Umfrage unter medizinischen Angestellten der Klinik für Anästhesiologie, Universitätsklinikum Würzburg, durchgeführt. Hierzu wurden validierte Tests zu den Domänen Depressivität, Ängstlichkeit, somatoforme Störungen, Burnout, Resilienz und Selbstmanagement verwendet und des Weiteren offene Fragen zu subjektiv belastenden, COVID-19-assoziierten Faktoren gestellt. Zusätzlich wurden Charakteristika der in den Umfragezeiträumen behandelten Patientinnen und Patienten sowie Informationen zum Krankenhausmanagement während der Pandemie erfasst.

**Ergebnisse:** In den beiden Erhebungszeiträumen wurden 24 bzw. 23 kritisch kranke COVID-19-Patientinnen und -Patienten behandelt. 87.5% bzw. 78.2% der Patientinnen und Patienten litten an einer moderaten bis schweren Form des adulten akuten Lungenversagens. Ab dem 6. März 2020 implementierte die Klinik eine streng hierarchische Notfall-Einsatzleitung, um tagesaktuell auf die Dynamik der Pandemie reagieren zu können, und die personellen Ressourcen wurden erhöht. Punktprävalenzen von Depressivität (13,6% und 12.8%) und Burnout (21,3% und 17.4%) bei den Studienteilnehmerinnen und -teilnehmern waren hoch. Die unmittelbare psychische Belastung war unabhängig von einer Exposition gegenüber SARS-CoV-2. Die Auswirkungen der Ausgangsbeschränkungen wurden von einer Mehrheit der Untersuchten als stark beeinträchtigend beschrieben. Hohe Resilienz schien protektiv zu wirken gegen Anzeichen von Depressivität, generalisierter Angst und Burnout.

**Schlussfolgerungen:** Im Verlauf der ersten Monate der Pandemie litt das medizinische Personal unter erhöhter psychischer Belastung verglichen mit früheren Vergleichsdaten sowohl der Allgemeinbevölkerung als auch von Personal auf Intensivstationen. Die allgemeinen Auswirkungen der Ausgangsbeschränkungen schienen hierbei einen größeren Einfluss gehabt zu haben als der tatsächliche Kontakt mit COVID-19-Patientinnen und -Patienten. Ausgeprägte Resilienz hatte einen positiven Effekt. Allerdings könnten anfällige Mitarbeiterinnen und Mitarbeiter unter Umständen gezielte Unterstützung benötigen. Zudem sind vorbeugende Maßnahmen gegen mögliche Nachwirkungen der Pandemie-Beschränkungen sowie alle Maßnahmen, die geeignet erscheinen, einen weiteren Lockdown zu verhindern, sinnvoll.

## Introduction

The COVID-19 pandemic is a challenge to healthcare systems worldwide, not only because of unprecedented resource demands, but also highly variable disease severity and progression, initial shortage of protective gear, lack of sufficient treatment, as well as adverse effects of countermeasures including lockdown (e.g. social isolation). Healthcare workers are particularly affected by increased workload, the anticipated overload of the healthcare system, and high risk of infection, which add to private challenges due to the lockdown. This obviously exacerbates the risks for occupational fatigue and burnout in ICUs.

Most studies on the psycho-social impact on employees in hospitals caring for COVID-19 patients are from China [1], [2], [3], [4]. They reported increased prevalence of depression and anxiety or even signs of posttraumatic stress disorder. A review of 14 studies on psychological distress in hospitals treating COVID-19 patients suggested a presence of clinical levels of depression and/or anxiety in 2.2–14.5% of staff members [5]. A more recent review including 28 observational studies suggested that “clear communication and support from the organization, social support and personal sense of control and coping ability are effective protective factors” [6]. In a nation-wide survey among frontline healthcare workers in Italy at around the same time as this work’s first survey, those who reported depression, anxiety, or stress (measured by the Depression Anxiety Stress Scale; DASS-21) were numerous: 28.5%, 21.4%, and 28.6%, respectively [7]. At this time, German healthcare facilities had a few weeks left to employ adaptive measures, as the pandemic broke out earlier in the affected southern European countries.

In this longitudinal study, we aimed to examine psychological well-being and identify possible protective and risk factors in healthcare providers in the department of anesthesiology of a high-volume ICU in a tertiary university hospital in Bavaria, Germany, which has been the state in Germany affected most severely by COVID-19 during the initial peak period.

## Methods

### Time and recruitment 

**Figure 1:**
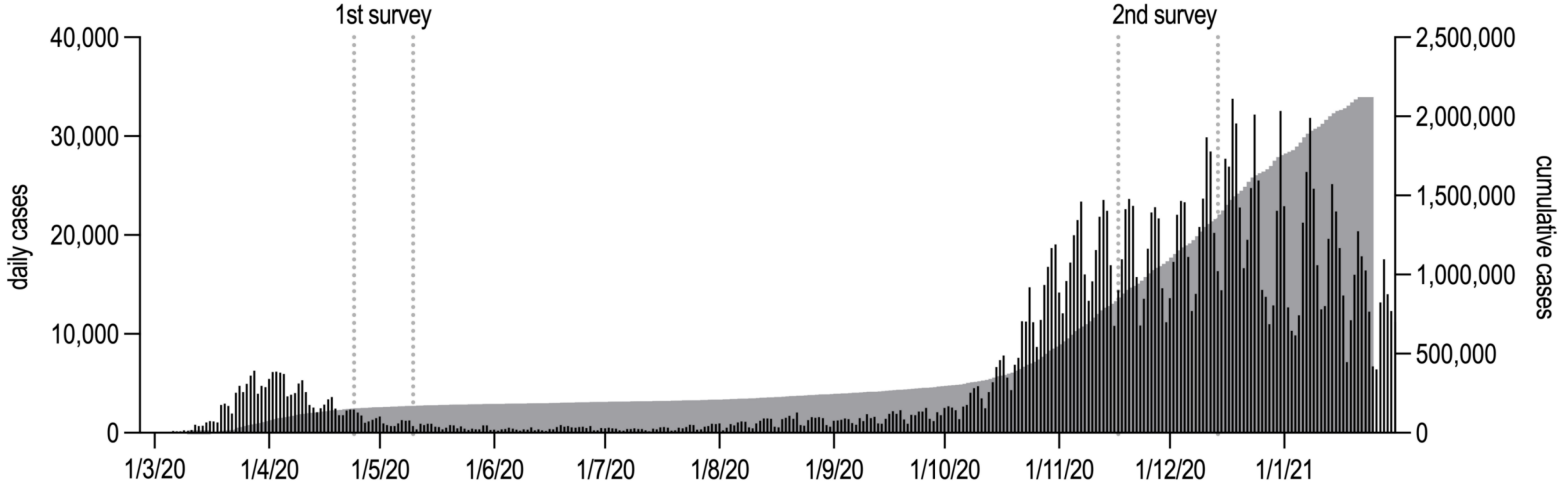
Daily (left y axis) and cumulative (right y axis) COVID-19 cases in Germany (all data from Robert Koch Institute). Both survey periods are marked by dotted lines.

The study is a longitudinal, hospital-based survey conducted in two waves from April 24 to May 10, and again from November 17 to December 14, 2020 (Figure 1 (Fig. 1)). Approval from the Ethics Committee of the University of Würzburg has been obtained (#73/20). Each time, all healthcare workers in the department of anesthesiology (about 300, half of them physicians) were asked to participate in this online survey study via email and printed flyers in order to reach out to as many eligible persons as possible. Informed consent was given prior to enrolment. Participants were allowed to terminate the survey at any time. The survey was anonymous, and confidentiality of information was assured. Those healthcare workers who participated in the first wave of the survey were assigned a pseudonymized code for their potential participation in the second wave. The codes were merely linked to an email address of the participant’s choosing and stored and managed by an independent administrator not otherwise engaged in the research study. Participants agreed to be contacted again after 3, 9 and 15 months or other points in time during this period, depending on the dynamics of new SARS-CoV-2 cases. We refrained from in-detail sample size calculations due to the limited number of potential participants and the fact that there was no controlled intervention as per study protocol which would have allowed for feasible estimates of a hypothesized effect.

For assessment of psychological burden in domains most likely affected by the challenge associated with the COVID-19 pandemic and effects of the lockdown (i.e. anxiety, depression, somatization, burnout), as well as possible protective factors (resilience, self-management, coping), well-established measures with good to excellent psychometric properties were used. Additional data was collected with customized items. For an overview of the collected items, see Table 1 (Tab. 1), and Attachment 1 for an extended description. Information of COVID-19 treated patients were obtained by chart review.

### Setting and COVID-19 specific measures before the first survey

The department of anesthesiology is a regional referral center for severe acute respiratory distress syndrome (ARDS) and specialized in extracorporeal membrane oxygenation. Its ICU comprises twelve beds, with an additional twelve beds at the surgical ICU.

To adapt to the (expected) workload before the first survey wave, medical and nursing staff was increased from 0.75 to 1.16 physicians/patient and from 2.67 to 2.98 nurses/patient. Medical students (0.22/patient) were added for supporting tasks (all personnel in a 3-shift system). In preparation for mass critical care, the University Hospital of Würzburg switched to a hospital incident command system (HICS) [8] from March 6, 2020 onwards. The HICS consists of a crisis unit with classic staff sections S1 to S6 (Table 2 (Tab. 2)). Head of the crisis unit was the medical director of the hospital with ultimate responsibility. HICS meetings took place at least once a day. Beyond staff and spatial planning of the mission, the supply shortage was a major focus of the HICS. Further measures in the department of anesthesiology included special trainings of residents and enhanced communication strategies.

### Statistical analysis

Means (M) and standard deviations (SD) were computed for continuous data; frequencies and percentages are provided for categorical variables. Pearson’s product momentum correlations were computed for data with normal distribution, Spearman rank correlations for skewed measures. Student’s t-tests (or Mann-Whitney U tests, as appropriate) were performed when comparing differences between groups. Multivariate general linear model analysis was used as an omnibus test for associations between protective and risk factors of psychological burden. Covariates of interest were centered, and the model was checked for interactions using repeated univariate analyses of variance. Lastly, simple linear regression analyses were used to obtain coefficient estimates for the individual dependent variables. For all tests, a significance level of α=.05 (two-tailed) was applied. All analyses were computed with available data. SPSS (IBM SPSS statistics 26) was used for all statistical tests.

## Results

### Study participants

In the first survey wave, a total of 87 participants responded with complete or near-complete datasets and were included in the analyses. In the second wave, 49 were included. Detailed characteristics of the participants are shown in Table 3 (Tab. 3).

### Status of the epidemic, characteristics of the COVID-19 patients and management during the first and second wave of the COVID-19 pandemic

On April 24, 2020, 150,383 people were tested positive for SARS-CoV-2 in Germany (all data from Robert Koch Institute) [9]. 5,321 people had died being SARS-CoV-2 positive. Until May 10, 169,218 people were SARS-CoV-2 positive and 7,395 had died after being tested positive for SARS-CoV-2 in Germany. By December 14, when the second wave of the survey ended, 1,366,494 people had been infected across Germany (Figure 1 (Fig. 1)).

During the first survey waves, 24 patients were treated for COVID-19-associated ARDS. According to the patients’ sequential organ failure assessment (SOFA) scores at the time of ICU admission, highest rates of mortality were to be expected (e.g. initial SOFA score of >11 was reported to be associated with 95% mortality) [10]. Therefore, these patients require a high degree of specialized care as represented in equally high scores in the Therapeutic Intervention Scoring System (TISS)-28 and the Simplified Acute Physiology Score (SAPS)-II. The majority (87.5%) of patients initially presented with moderate to severe ARDS. Around two thirds of the patients required extracorporeal membrane oxygenation, and one third relied on renal replacement therapy. Mean duration of ventilation was almost three weeks. 37.5% died while in the ICU. During the second wave, 23 patients with rather similar characteristics were treated. However, the percentage of patients with severe ARDS was markedly higher (56.55), leading to an increase in mortality (47.6%). For detailed patient characteristics, see Table 4 (Tab. 4). In summary, COVID-19 patients treated in the ICU were severely sick, requiring highest levels of ICU care.

### Increased psychological impact in healthcare professionals during the COVID-19 pandemic independent of immediate exposure

In our cohort, initially 13.6% of participants reached a score of 3 or more in the PHQ-2, which is the established cut-off to indicate likely presence of major depression (Table 3 (Tab. 3)) [11]. Similarly, 12.5% of participants exceeded a cut-off of 10 on the GAD-7, suggesting the likely presence of generalized anxiety [12]. In our two-item version of the SSS-8 [13], the majority of the participants showed no/minimal or low symptoms (51.1% and 26.1%, respectively) for somatization disorders (analysis adjusted to scale abbreviation). In the MBI, 21.3% of participants reported signs of burnout [14], [15]. Half of the participants reported high trait resilience in the RS-13 [16], whereas 27.3% showed low and 21.6% moderate trait resilience. In the FERUS-26 questionnaire of resources and self-management capabilities [17], participants in general reached normal scores. The mean of the T-transformed overall score was 49.6±9.1, most subscales showed similar results (Attachment 1, Table 1 ). Only in the subscales ‘motivation for change’ and ‘self-verbalization’, participants scored up to 1 SD below the T-norm of 50 (40.0±8.1 and 44.3±10.2, respectively).

Psychological test scores from the second wave of the survey were overall very similar to those from the first wave (Table 3 (Tab. 3)). Three noteworthy exceptions were: Firstly, the percentage of participants who met the cut-off for major depression in the PHQ-2 remained stable over time; it dropped from 25.0% to 0% in nursing staff. Secondly, the severity of somatic afflictions increased, with 26.3% more participants presenting with moderate symptoms. Thirdly, resilience decreased over the course of the pandemic. 19.6% less employees had high resilience in the RS-13.

**Figure 2:**
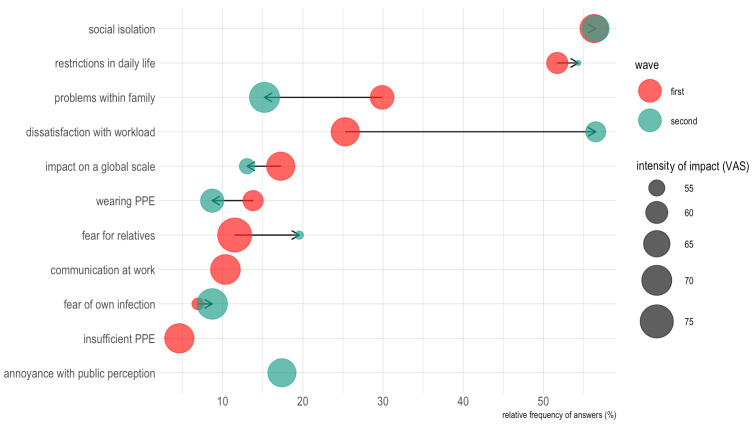
Perceived impact of pandemic-related factors on personal well-being. Relative frequency denotes the percentage of participants who named the respective issue among their top 3 most distressing factors. Dot sizes correspond to the average VAS rating this particular item was assigned by the participants.

The most distressing aspects of their life in the ongoing pandemic reported by the participants during the initial phase of the pandemic included social isolation (56%), limitations in their everyday life and activities (sports, vacation, etc.; 52%), and problems within the family (e.g. childcare or domestic conflicts 30%, see Figure 2 (Fig. 2)). Some of these conflicts resolved over time: the need to wear PPE, concerns regarding the large-scale impact of the pandemic, and problems within the family. Fear of PPE supply shortages and insufficient communication by the employer were not mentioned anymore. However, social isolation remained at its high level, and dissatisfaction with workload doubled its impact to more than 55%.

Increased sum scores in the PHQ-2 during the first wave were associated with a) a higher perceived impact of stress factors (r=.24), b) higher fear of COVID-19 sequelae (r=.21), and c) concerns about friends and family (r=.31) during the first wave. GAD-7 sum scores were also positively correlated with these factors respectively (r_a_=.37, r_b_=.17, r_c_=.42). Both the SSS-2 sum score (r_a_=.13, r_b_=.27, r_c_=.14) and the overall MBI score (r_a_=–.02, r_b_=–.06, r_c_=.07) were only weakly correlated with any of the above outcomes. Due to the limited number of participants in the second wave and an overall high consistency of the results, we refrained from such analyses with those data.

### Different psychological well-being between professions unrelated to COVID-19 exposure

In the PHQ-2 and GAD-7, the percentage of nurses with moderate/severe symptoms was doubled compared to physicians during the first wave (Table 3 (Tab. 3)). Percentages of participants indicating burnout symptoms were more evenly distributed. Protective factors like trait resilience were likewise evenly distributed across professions. After categorization, high trait resilience was observed more often in physicians than in nurses; similarly, self-management capabilities were significantly higher in physicians than nurses (p=0.049, Bonferroni-adjusted post-hoc analysis). There were no significant differences in the subscales (all p>0.05). Again, due to the limited number of participants, we did not perform such analyses between subgroups for the second wave of data.

We next hypothesized that contact with a SARS-CoV-2 positive/COVID-19 patient either professionally of privately would lead to less psychological well-being. However, neither PHQ-2, GAD-7, MBI, nor SSS-2 were significantly different between groups with and without exposure. Likewise, trait resilience and self-management resources were similar between groups with and without SARS-CoV-2 contact during the first survey period.

### Trait resilience as an important protective factor independent of COVID-19 exposure

We finally sought to identify potential predictors/protective factors for psychological distress. Due to previous findings in this field, we hypothesized that trait resilience might be an appropriate measure. In correlation analyses, we found trait resilience to be significantly negatively correlated with several indicators of psychological distress (sum score for GAD-7: r=–0.47, PHQ-2: r=–0.57, SSS-2: r=–0.29, MBI: r=–0.43, all p<0.01). Next, we entered these variables into a multivariate general linear model. We used the RS-13 sum score as the fixed factor, GAD-7, PHQ-2, SSS-2, and MBI as dependent variables, and initially also included sociodemographic features (age, gender, profession, job experience) and past contact with COVID-19 patients as covariates. Since univariate ANOVAs revealed that SSS-2 scores were significantly influenced by the ‘age x job experience’ interaction (F(1, 72)=11.91, p<0.001; all other interaction terms: p>0.05), this interaction was included in the regression as well. As an omnibus test, this analysis turned out significant for trait resilience as a global predictor of psychological distress (for detailed results, see Attachment 1, Table 2 ), but not for any of the covariates (all p>0.16). In subsequent linear regressions, higher RS-13 sum scores predicted lower scores of PHQ-2, GAD-7, SSS-2, and MBI.

## Discussion

This longitudinal survey analyzed psychological well-being of healthcare workers in the department of anesthesiology and critical care of a tertiary university hospital in Germany treating severely sick COVID-19 patients during two observation periods within the ongoing COVID-19 pandemic. Despite an implemented comprehensive disaster response regimen, symptoms of depression and generalized anxiety were higher than reported in the general population, and one fifth of healthcare workers reported symptoms indicating burnout. A majority, however, was more burdened by consequences of the lockdown than by work-related strains. Moreover, we did not collect true baseline data before the pandemic started, so we cannot estimate any possible effects of the (potentially) protective measures taken by the hospital administration. Psychological well-being was significantly dependent on trait resilience, and no effect of SARS-CoV-2 exposure could be determined in the current analysis. This is different from previous findings from a similar study in Germany [18] and a review of studies from China [19], where direct exposure tended to affect psychological burden.

### Inferior psychological well-being of healthcare professionals compared to the general population or healthcare professionals before the pandemic

The prevalence of depressive symptoms in the general population in Germany using the PHQ-9 is 8.1% [20], while 5.7% of physicians and 3.1% of emergency medicine physicians reported depressive symptoms above established cut-offs [21], [22]. In the US, 11% and 18% of ICU nurses suffered from depressive or anxious symptomatology, respectively [23]. The prevalence of anxiety in physicians was 31% in a Chinese study [24]. Consequently, in our cohorts, the prevalence of these symptoms at both investigated points in time was higher than in previous studies in Germany, but well in line with findings from the US and China. We can only speculate whether nurses with depressive symptoms chose to participate in the surveys.

Burnout varies largely between countries and professions, which is also due to inconsistent definitions [25]. In Germany, the prevalence in the general population is 4.2%, but in intensive care physicians in 2016, the prevalence was 8.2% [21], and in the US as high as 24.1% [26]. In our study, the current proportion was more than doubled compared to published data on German ICU physicians. Burnout for nurses also varies greatly. Representative prevalence estimates based on MBI assessments range from 40% for Spanish nurses in primary health care [27] to 80% for ICU nurses in the US [23]. A survey conducted in five German hospitals reports lower scores than the US study on the three MBI subscales [28], yet even the mean scores exceed cut-offs for moderate to high levels of burnout [15], [29]. In comparison, reported burnout symptoms in our study were much more frequent during the first two peaks of the COVID-19 pandemic across professions despite increases in resources and personnel.

### Comparable psychological impact on healthcare workers in other countries

Frontline nurses and physicians treating COVID-19 patients are exposed to an estimated 3.4-fold increased risk for SARS-CoV-2 infection based on data from China [30]. SARS-CoV-2-specific sources of burnout may include feelings of vulnerability or loss of control and concerns about one’s own health, spread of virus to others, health of family members, and others. Taking into account the use of different instruments, a high percentage of depression (30.6% by PHQ-4), anxiety (28.0% by PHQ-4), and distress (20.1% by K6) was found in Iran [3] as well as China (Wuhan): depression (50.3%, thereof 12.6% with moderate/severe depression), anxiety (48.4%/11.4%), and distress (74.8 %/39.1%) at the end of January 2020 [3]. However, another multinational study reported only low levels of moderate/severe depression (5.3%) or anxiety (8.7%) [31]. A recent rapid review based on healthcare workers from China reported 14% to 15% clinically relevant symptoms for depression, 12% to 24% for anxiety, and 30% to 39% for psychological distress [30], comparable with our cohort. This might suggest that the extent of the pandemic might not be the critical factor when comparing Wuhan/China and Bavaria/Germany.

### Protective properties in participants and measures taken to prevent psychological impairment

For psychological distress to become relevant, stressing factors must be powerful enough to overcome an individual’s protective resources [32]. To balance out the increased stress due to COVID-19 consequences, workforce resources were increased in anticipation of rising patient numbers before this study was initiated. Existing ICU capacities were at all times sufficient. Nevertheless, individual COVID-19 patients had higher demands for medical personnel than regular patients.

The participating healthcare professionals showed close-to-average self-management capabilities as indicated by the FERUS-26 score. Nursing staff had slightly lower scores during the first survey period. This difference was not detectable in the second survey. Also, trait resilience was well developed in participants, with higher trait resilience in physicians compared to nurses and non-medical staff. Matched to the general population, physicians reached high trait resilience more frequently than expected, while nurses and especially non-medical personnel were under-represented in this category [16]. Bearing in mind all limitations inherent to this study, resilience as indicated by the RS-13 score was significantly lower in individuals exposed to COVID-19 patients during the second survey when compared to the corresponding subgroup of the first period (p=0.012, Mann-Whitney U test, Table 5 (Tab. 5)). Trait resilience is known to protect against burnout [23], [33] and was significantly (negatively) correlated with most of the test scores in our cohort from the first survey. In a multivariate regression model, trait resilience substantially predicted the risk for developing signs of depression and general anxiety during the first survey period. Although trait resilience was not distributed entirely equally among genders and professions, both these variables as well as age and job experience or the exposition to COVID-19 were not found to significantly moderate its effect on said conditions. Due to the lower response rate during the second survey period, we were not able to perform all the in-depth analyses like we did with the first set of data. Descriptive analyses, however, do not suggest substantial changes in psychological burden among healthcare workers.

### Limitations

During the first wave of the study, less than 50% of all healthcare workers in the department responded, and the percentage of nurses was even lower. Thus, we cannot exclude a certain selection bias. This is even more the case for the second survey, where only half the number of participants could be included. The number of participants who completed both surveys was too small for any reasonable evaluation. Given the context of our study, where participants were surveyed regarding their immediate working environment, social-desirability bias has to be taken into consideration as well. Especially in our monocentric setting and the relatively small group of possible participants, it is possible that traits like resilience and self-management capabilities were overestimated, whereas psychological burden might have been downplayed to some extent. Furthermore, our data are based on a single center. We opted to neither differentiate between private and professional exposure to SARS-CoV-2 nor between healthcare workers on the ICU and in the operating room. Furthermore, no data before the pandemic were available. Because of the rapidly changing COVID-19 situation in Germany and Europe, mental health symptoms of healthcare workers could become more or less severe. Thus, long-term psychological implications of this population are important for further investigation.

## Conclusions

Psychological well-being is impaired in healthcare professionals treating severely affected COVID-19 patients in Germany. General restrictions due to the lockdown may have imposed more burden than SARS-CoV-2 exposure. Apart from work-related adaptions, strengthening self-management and resilience could prevent further psychological impairment and diseases. This calls for special interventions to prevent depression, anxiety disorders, and burnout. We here propose a multi-dimensional approach which seeks to reduce the impact of stress and tries to strengthen each individual’s coping resources. In our study, direct COVID-19 exposure did not seem to add to the psychological burden in healthcare workers as opposed to previous findings [19]. This may indicate that the measures taken at our hospital previous to this study may have contributed to reducing psychological strain on healthcare workers. Finally, our data suggest that COVID-associated restrictions may be even more important than exposure to infected individuals.

## Abbreviations

- ARDS: Acute respiratory distress syndrome
- COVID-19: Coronavirus disease 2019
- FERUS: Questionnaire on resources and self-management capabilities [Fragebogen zur Erfassung von Ressourcen und Selbstmanagementfähigkeiten]
- GAD-7: General anxiety disorder (questionnaire with 7 items)
- HICS: Hospital incident command system
- ICU: Intensive care unit
- M: Mean
- MBI: Maslach burnout inventory
- PHQ-2: Patient health questionnaire (2 items)
- RS-13: Resilience scale (13 items)
- SAPS-II: Simplified acute physiology score II
- SARS-CoV-2: Severe acute respiratory syndrome coronavirus 2
- SD: Standard deviation
- SOFA: Sequential organ failure assessment
- SSS-8: Somatic symptom scale (8 items)
- TISS-28: Therapeutic intervention scoring system (28 items)

## Notes

### Trial registration

The trial has been registered in the German registry for clinical studies (https://www.germanctr.de; registration number DRKS00021649).

### Authors’ contributions

BS and SMS contributed equally.

### Acknowledgments

The authors thank all staff for their participation. Assistance with this study: We acknowledge Udo Selig from the Institute of Clinical Epidemiology and Biometry (ICE-B, Würzburg University) for implementation of the online questionnaire.

### Competing interests

The authors declare that they have no competing interests.

## Supplementary Material

Supplementary material

## Figures and Tables

**Table 1:**
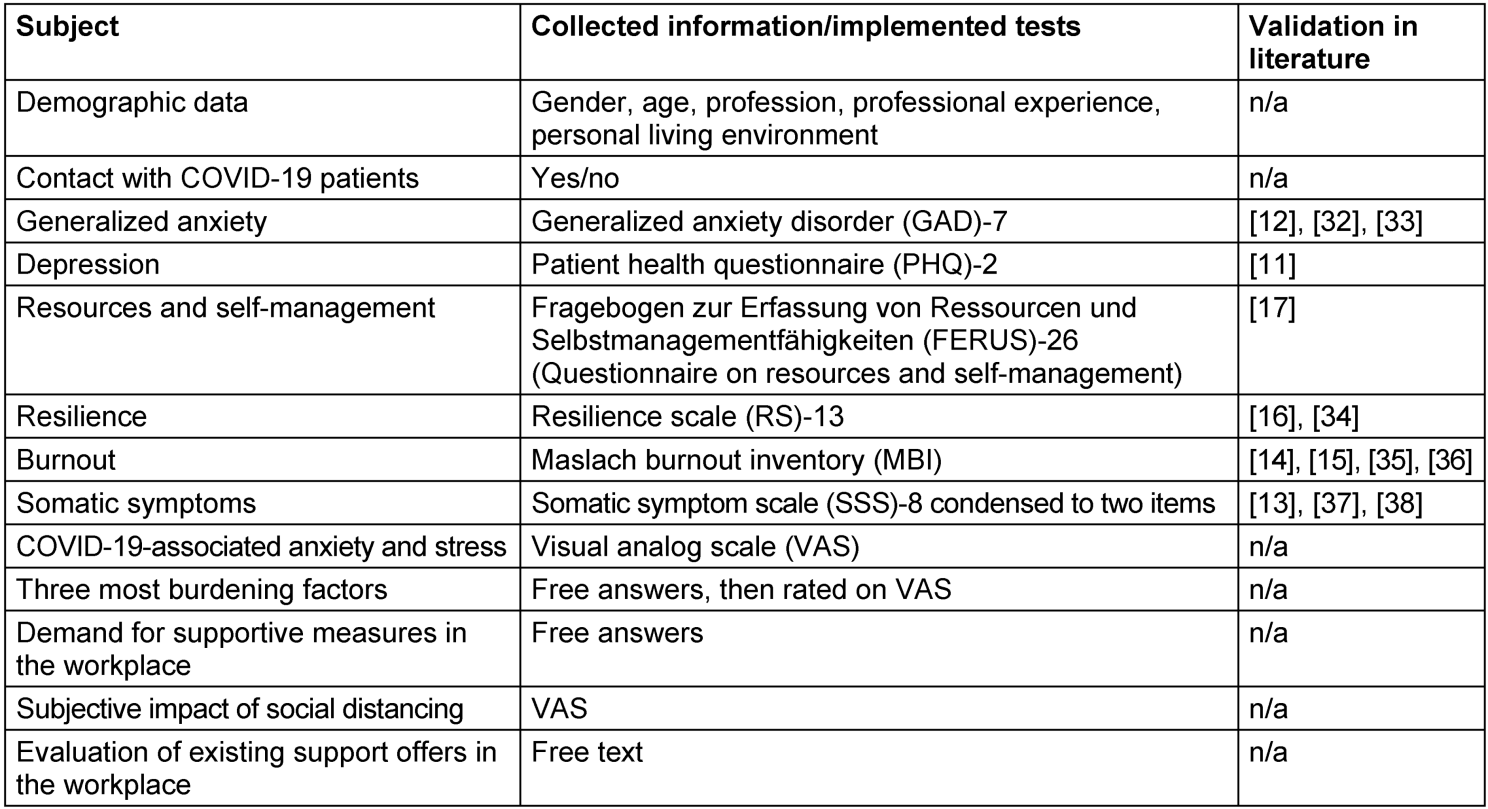
Items of the questionnaire

**Table 2:**
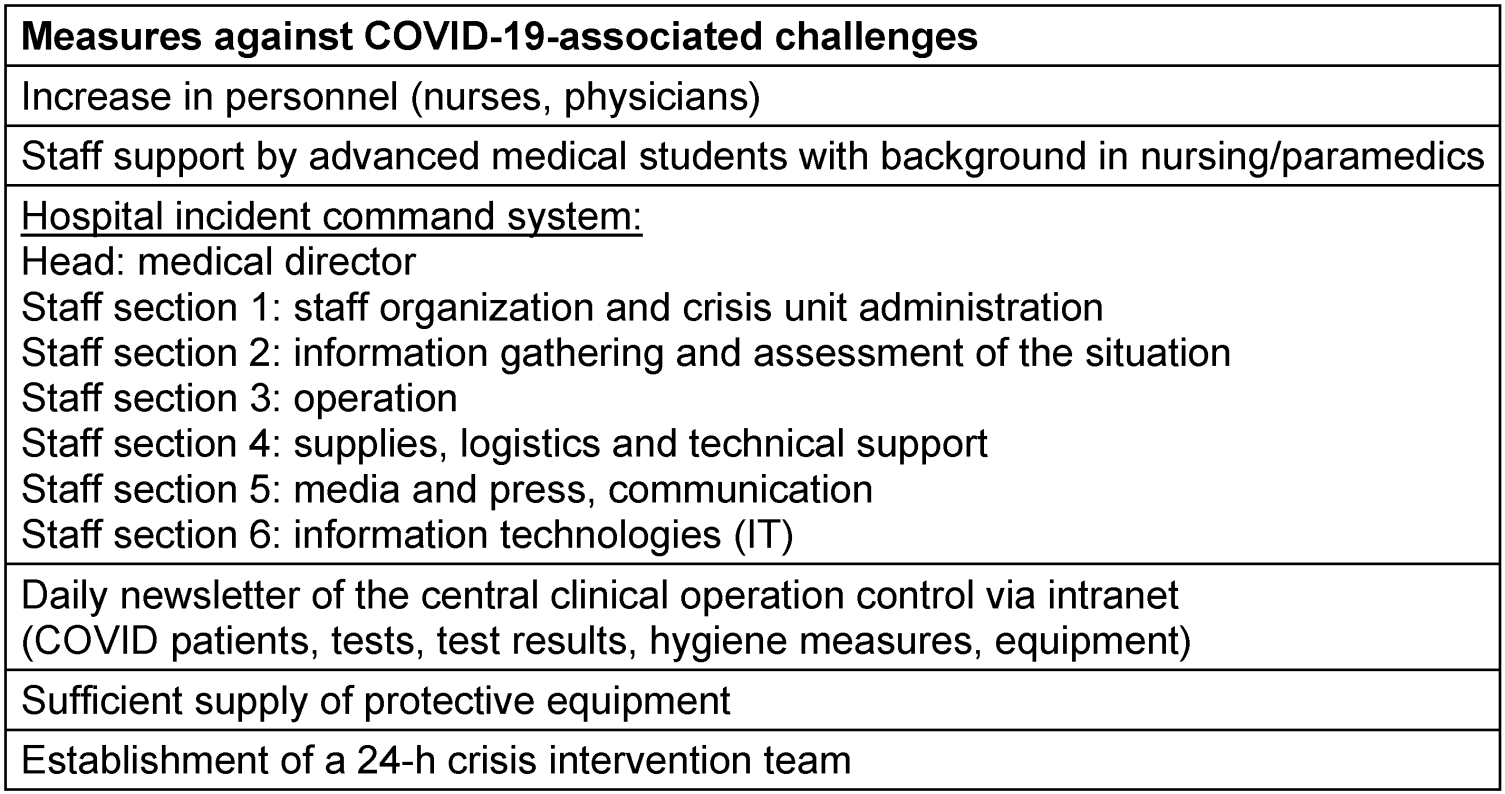
Adaptation of work conditions

**Table 3:**
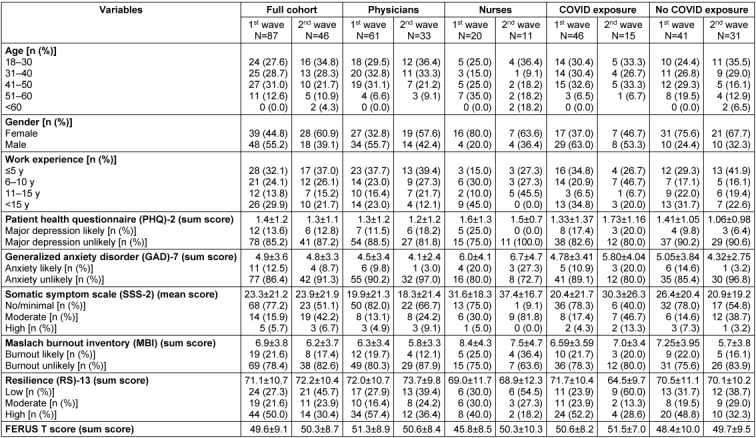
Demographics and psychological well-being is independent of COVID-19 exposure

**Table 4:**
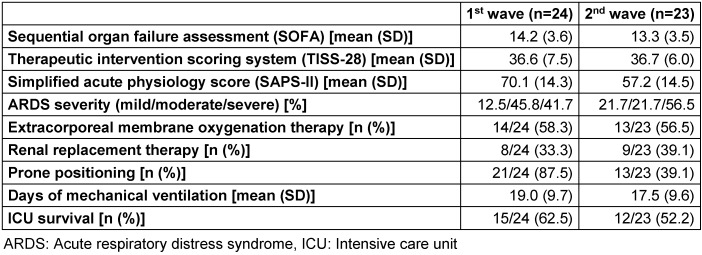
Patient characteristics

**Table 5:**
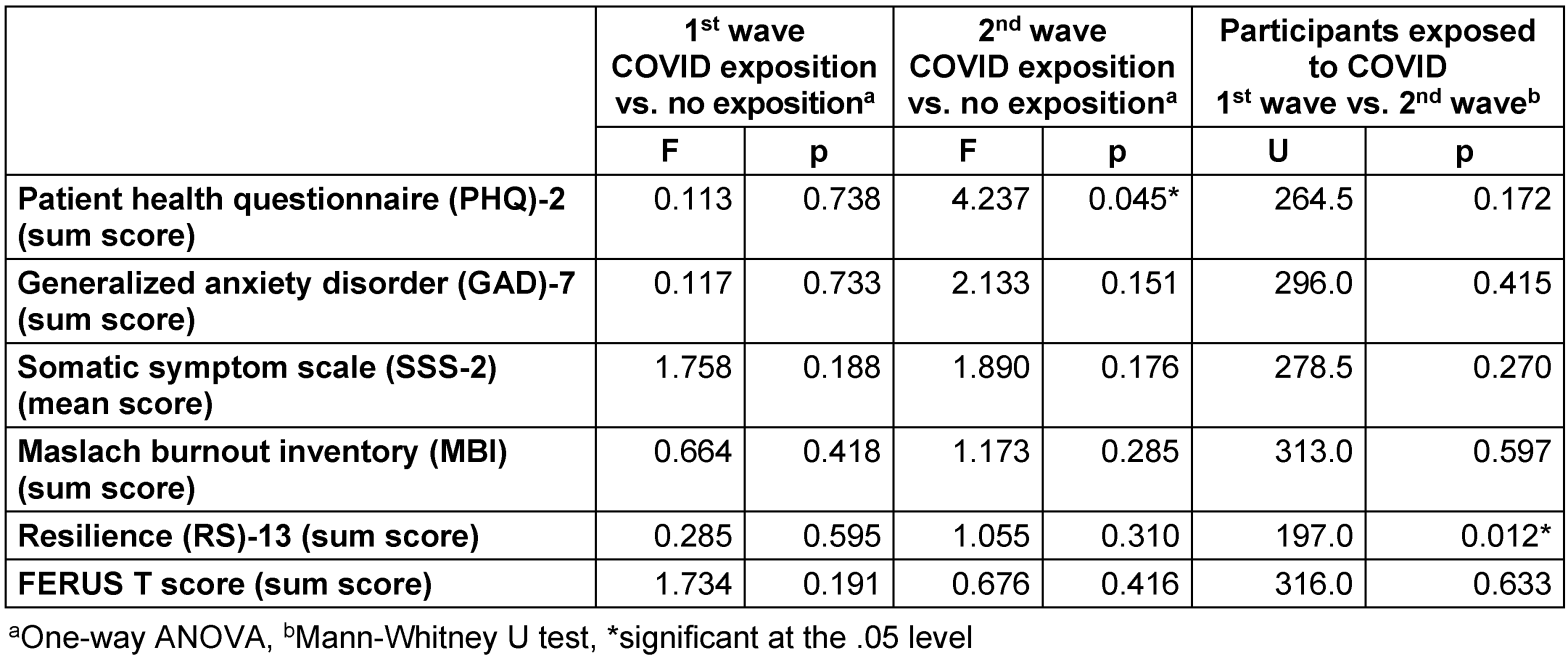
Comparison of psychometric test results depending on COVID-19 exposure and over time
