# Supplementary material for: Impaired psychological well-being of healthcare workers in a German department of anesthesiology is independent of immediate SARS-CoV-2 exposure – a longitudinal observational study
Source: Ger Med Sci. 2021 Sep 1;19:Doc11. doi: 10.3205/000298 (PMC8422798; doi:10.3205/000298)
Supplement: Supplementary material [file GMS-19-11-s-001.pdf]

Supplementary material

Measures – extended description

Demographic data were self-reported by the participants including occupation (physician, nurse, or other), sex (male, female, or diverse), age (18–25, 26–30, 31–40, or >40 years), living status. Participants were asked whether they had – to their own knowledge – work-related or private contact to patients with COVID-19 disease.

The Generalized Anxiety Disorder 7 (GAD-7) [1] and the Patient Health Questionnaire-2 (PHQ-2) [2] are self-report questionnaires derived from the Patient Health Questionnaire for efficient screening and severity measuring of generalized anxiety disorder (GAD) and depression respectively.

The seven items of the GAD-7 capture frequency of occurrence within the past two weeks for the most important DSM-IV criteria for GAD diagnosis. A cut-off score of 10 applied to the sum score achieves sensitivity of 89% and specificity of 82%; internal consistency (Cronbach's alpha=.89) and test-retest reliability (intraclass correlation coefficient [ICC]=.83) are high [1]. The GAD-7 has been validated in a representative German sample [3]. Norm derived from this sample revealed an overall mean sum score of M=2.95 (SD=3.41). Data is also available for patients with a clinical diagnosis (GAD: M=14.0, panic disorder: M=12.5, posttraumatic stress disorder: M=12.0) [4].

The two-item PHQ.2 measures frequency of depressed mood and anhedonia over the past two weeks. A cut-off score of three, applied to the sum score, achieves sensitivity of 87% and specificity of 78%; internal consistency (Cronbach's alpha=.83) is high, and the PHQ-2 has been shown to be sensitive to change [2]. Comparison scores for both healthy individuals (M=1.4, SD=1.3) and patients with a clinical diagnosis are available (major depression: M=4.7, SD=1.5; any depressive disorder: M=3.4, SD=1.7) [2].

The German Fragebogen zur Erfassung von Ressourcen und Selbstmanagementfähigkeiten (Resources and Self-Management Skills Questionnaire, FERUS) [5] measures health-related resources and self-management

via 26 items (Likert-scale from 1=not accurate to 5=very accurate) on seven subscales (motivation to change, self-monitoring, active and passive coping, self-efficacy, self-verbalization, hope, and social support). The scales are summarized in an overall self-management score with higher scores representing superior resources. The FERUS-26 has been sufficiently validated. Retest-reliability of all scales is satisfactory or good (0.66 to 0.86), internal consistency is good to excellent (0.86 to 0.93) [5]. The test comes with validated reference data for T transformation which we used in this manuscript.

The German Resilience scale RS-13 [6] is a well-established self-report questionnaire designed to assess trait resilience with 13 statements that are rated from ‘1=no, I do not agree’ to ‘7=yes, I completely agree’. Higher sum scores reflect a person’s global capability to adapt positively to challenging conditions [7]. The scale has been validated in several studies, has high internal consistency (Cronbach’s alpha=.90), and offers reasonably high retest reliability (.61) [6].

The Maslach burnout inventory consists of 22 statements answered in a ‘yes/no’ format. The sum score reflects an individual’s experience of occupational burnout [8]. The scale offers strong reliability (≥.76), retest reliability ranging from .82 for a few weeks to .54 for 1 year, and has been validated across different work divisions [9], [10].

For economic assessment of somatic symptom burden, we proportionately condensed the well-validated [11], [12] Somatic Symptom Scale-8 (SSS-8) with high internal consistency (Cronbach’s alpha=.81) that is sensitive to change [13] into a 2-item screener reflecting the two most prominent domains of somatic symptom disorder (i.e. pain and gastro-intestinal symptoms).

Additional items were generated for assessment of duration and intensity of contact with COVID-19 patients according to the criteria of the Robert Koch Institute as of April 24, 2020, COVID-19-associated anxiety and stress experiences (visual analog scale, VAS), and effects of ‘social distancing’ (VAS). Open questions were used to record the three currently most stressful factors (associated weighting via VAS), measures that could help to cope with stress in the workplace, and usage/knowledge of support offers established by the UKW.

References

1. Spitzer RL, Kroenke K, Williams JB, Löwe B. A brief measure for assessing generalized anxiety disorder: the GAD-7. Arch Intern Med. 2006 May;166(10):1092-7. DOI: 10.1001/archinte.166.10.1092
2. Löwe B, Kroenke K, Gräfe K. Detecting and monitoring depression with a two-item questionnaire (PHQ-2). J Psychosom Res. 2005 Feb;58(2):163-71. DOI: 10.1016/j.jpsychores.2004.09.006
3. Löwe B, Decker O, Müller S, Brähler E, Schellberg D, Herzog W, Herzberg PY. Validation and standardization of the Generalized Anxiety Disorder Screener (GAD-7) in the general population. Med Care. 2008 Mar;46(3):266-74. DOI: 10.1097/MLR.0b013e318160d093
4. Kroenke K, Spitzer RL, Williams JB, Monahan PO, Löwe B. Anxiety disorders in primary care: prevalence, impairment, comorbidity, and detection. Ann Intern Med. 2007 Mar;146(5):317-25. DOI: 10.7326/0003-4819-146-5-200703060-00004
5. Jack M. FERUS – Fragebogen zur Erfassung von Ressourcen und Selbstmanagementfähigkeiten. Z Kl Psych Psychoth. 2007;37(3):213-4.
6. Leppert K, Koch B, Brähler E, Strauß B. Die Resilienzskala (RS) - Überprüfung der Langform RS-25 und einer Kurzform RS-13. Klin Diagnostik Evaluation. 2008;1:226-43.
7. Luthar SS, Cicchetti D, Becker B. The construct of resilience: a critical evaluation and guidelines for future work. Child Dev. 2000 May-Jun;71(3):543-62. DOI: 10.1111/1467-8624.00164
8. Maslach C, Jackson SE, Leiter MP. The Maslach burnout inventory manual. 2nd edition. Palo Alto: Consulting Psychologists Press; 1996.
9. Iwanicki EF, Schwab RL. A cross validation study of the Maslach Burnout Inventory. Educ Psychol Meas. 1981;41(4):1167-74. DOI: 10.1177/001316448104100425
10. Gold Y. The factorial validity of the Maslach Burnout Inventory in a sample of California elementary and junior high school classroom teachers. Educ Psychol Meas. 1984;44(4):1009-16. DOI: 10.1177/0013164484444024

11. Gierk B, Kohlmann S, Kroenke K, Spangenberg L, Zenger M, Brähler E, Löwe B. The Somatic Symptom Scale–8 (SSS-8): A Brief Measure of Somatic Symptom Burden. JAMA Intern Med. 2014;174(3):399-407. DOI: 10.1001/jamainternmed.2013.12179
12. Zijlema WL, Stolk RP, Löwe B, Rief W; BioSHaRE, White PD, Rosmalen JG. How to assess common somatic symptoms in large-scale studies: a systematic review of questionnaires. J Psychosom Res. 2013 Jun;74(6):459-68. DOI: 10.1016/j.jpsychores.2013.03.093
13. Gierk B, Kohlmann S, Hagemann-Goebel M, Löwe B, Nestoriuc Y. Monitoring somatic symptoms in patients with mental disorders: Sensitivity to change and minimal clinically important difference of the Somatic Symptom Scale – 8 (SSS-8). Gen Hosp Psychiatry. 2017 Sep;48:51-5. DOI: 10.1016/j.genhosppsych.2017.07.002

FERUS domain	Full cohort mean (SD)	Physicians	Nurses	Other	COVID contact	No COVID contact
Overall score	49.6 (9.1)	51.3 (8.9)	45.8 (8.5)	43.4 (6.9)	48.4 (10.0)	50.6 (8.2)
Motivation for change	40.1 (8.1)	39.8 (8.0)	40.0 (8.8)	42.8 (8.5)	40.1 (9.1)	49.4 (9.6)
Coping	50.4 (9.2)	52.0 (9.4)	47.1 (7.4)	44.2 (8.2)	49.4 (9.6)	51.2 (8.8)
Self-efficacy	52.7 (9.2)	54.4 (8.7)	49.3 (9.4)	44.4 (7.7)	51.5 (9.7)	53.7 (8.8)
Self-verbalization	44.3 (10.2)	46.0 (10.1)	39.9 (9.8)	40.8 (7.7)	44.1 (11.5)	44.5 (9.1)
Hope	52.0 (9.0)	52.9 (8.4)	50.0 (10.8)	49.6 (8.8)	49.9 (9.7)	53.9 (8.0)
Social support	50.8 (8.7)	50.9 (8.4)	50.5 (9.1)	50.4 (13.4)	50.7 (9.3)	50.9 (8.4)

Supplementary Table 1: T-scores in FERUS sub-domains (n=86)

Dependent variables	**General linear model** Wilks λ=0.02 F(148, 162.04)=1.80 p<0.001	Linear regression
GAD-7	R^2=0.67	β=–0.47 **
PHQ-2	R^2=0.71	β=–0.57 **
SSS-2	R^2=0.63	β=–0.29 **
MBI	R^2=0.80	β=–0.43 **

Supplementary Table 2: Results of general linear modeling of RS-13 as a predictor of psychological burden. GAD: general anxiety disorder, PHQ: patient health questionnaire, SSS: somatic symptom scale, MBI: Maslach burnout inventory; **p<.01
